# Association of arterial stiffness with all-cause and cause-specific mortality in the diabetic population: A national cohort study

**DOI:** 10.3389/fendo.2023.1145914

**Published:** 2023-03-08

**Authors:** Cun Liu, Huachun Pan, Fanliang Kong, Shumin Yang, Quazi T. H. Shubhra, Dandan Li, Siwei Chen

**Affiliations:** ^1^ Department of Hypertension and Plateau Disease, Qinghai Province Cardiovascular and Cerebrovascular Disease Specialist Hospital, Qinghai, China; ^2^ College of Veterinary Medicine, Huazhong Agricultural University, Wuhan, China; ^3^ Department of Cardiology and Pneumology, University Medical Center of Göttingen, Georg-August University, Göttingen, Germany; ^4^ German Centre for Cardiovascular Research (DZHK), Göttingen, Germany; ^5^ State Key Laboratory of Agriculture Microbiology, College of Veterinary Medicine, Huazhong Agricultural University, Wuhan, China; ^6^ Stomatology Hospital of Guangzhou Medical University, Guangzhou Medical University, Guangzhou, China; ^7^ Translational Medicine Engineering Research Center of Guangdong Province, Foshan First People’s Hospital, Foshan, China; ^8^ Department of Nursing, Zaozhuang Vocational College, Shandong, China; ^9^ Department of Cardiovascular Medicine, The Third Hospital of Nanchang, Jiangxi, China

**Keywords:** estimated pulse wave velocity, diabetes mellitus, national health and nutrition examination survey, all-cause mortality, cardiovascular disease, mortality

## Abstract

**Background:**

Estimated pulse wave velocity (ePWV) has been proposed as a potential alternative to carotid-femoral pulse wave velocity to assess the degree of aortic stiffness, and may predict cardiovascular disease (CVD) outcomes and mortality in the general population. However, whether arterial stiffness estimated by ePWV predicts all-cause and cause-specific mortality in patients with diabetes mellitus (DM) has not been reported.

**Methods:**

This was a prospective cohort study with data from the National Health and Nutrition Examination Survey (NHANES) from 1999 to 2014 and followed up until the end of December 2019. 5,235U.S. adults with DM (age≥20years) were included in the study. Arterial stiffness was estimated by ePWV. Survey-weighted Cox proportional hazards models were performed to assess the hazard ratios (HRs), and 95% confidence intervals (CIs) for the associations of ePWV with all-cause and cause-specific mortality. Meanwhile, the generalized additive model was used to visually assess the dose-dependent relationship between ePWV and mortality. As a complementary analysis, the relationship between mean blood pressure (MBP) and risk of mortality was also examined. Multiple imputations accounted for missing data.

**Results:**

For the 5,235 DM patients, the weighted mean age was 57.4 years, and 51.07% were male. During a median follow-up period of 115 months (interquartile range 81-155 months; 53,159 person-years), 1,604 all-cause deaths were recorded. In the fully adjusted Cox regression model, every 1 m/s increase in ePWV was associated with 56% (HR 1.56; 95% CI, 1.44 to 1.69) increase in the risk of all-cause. In addition, a nonlinear relationship between ePWV and all-cause mortality was observed (P for non-linear=0.033). Similar results were obtained after subgroup analysis and multiple imputations. Besides, the risk of most cause-specific mortality, except for accident and renal disease-specific mortality, increased from 53% to 102% for every 1 m/s increase in ePWV.

**Conclusions:**

In the diabetic population, ePWV is independently associated with all-cause and most cause-specific mortality risks. ePWV may be a useful tool for assessing mortality risk.

## Graphical abstract

41,597 US adults enrolled in NHANES 1999-2014, with follow-up until the end of December 2019. After excluding non-diabetics, pregnant women, people with cancer and people without ePWV data, 5235 diabetics were finally included in the analysis. Arterial stiffness was estimated by ePWV. The results showed a non-linear positive association between ePWV and the risk of all-cause mortality(P for non-linear=0.033). When ePWV was<7.2m/s, the risk of all-cause mortality increased by 2.1-fold (HR 3.10; 95% CI, 1.54 to 6.25; P=0.002) with an increase in ePWV of 1 m/s; when ePWV was≥7.2m/s, the risk of all-cause mortality increased by 54%(HR 1.54; 95% CI, 1.44 to 1.64; P<0.001). In addition, the risk of cause-specific mortality increased from 53% to 102% for every 1m/s increase in ePWV. Graphical abstract created with BioRender.com.NHANES, National Health and Nutrition Examination Survey. ePWV, estimated pulse wave velocity.

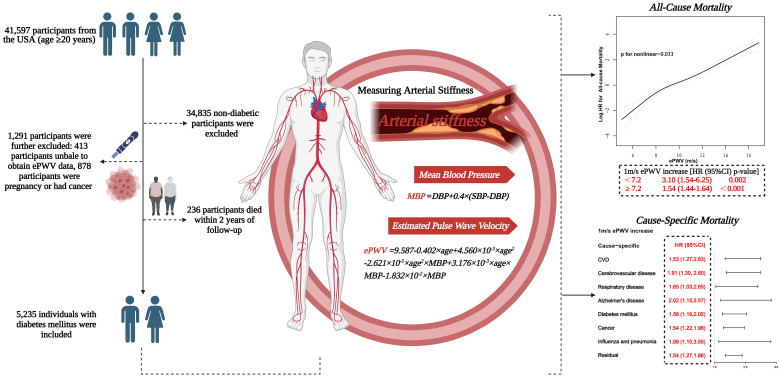

## Introduction

Atherosclerosis is a widespread chronic inflammatory disease of the arterial wall and manifests mainly as lesions and plaque accumulation in the intimal layers of the arterial wall (1). Numerous studies have shown that diabetes and atherosclerosis coexist in multiple pathophysiological pathways, and diabetes mellitus (DM) accelerates the progression of atherosclerosis (1, 2). Plaque formation in a large artery (e.g., the aorta) causes the narrowing of the lumen and stiffening of the arterial wall and exposes the target organ to potentially damaging hemodynamic forces (3). Arterial stiffness is a clinical hallmark of arterial aging. Measures of aortic stiffness can reflect the severity of vascular stiffness and capture the vessel’s age. This further provides additional insight into cardiovascular disease (CVD) risk beyond the traditional CVD risk factors (4). Currently, carotid-femoral pulse wave velocity (cfPWV) is the gold standard and a widely used method in epidemiological studies to assess central arterial stiffness (5, 6). However, the measurement of cfPWV requires specific equipment, specialized technical proficiency, and special procedures that limit its clinical application.

For this reason, a potential alternative to cfPWV, the estimated pulse wave velocity (ePWV), has been proposed (7). ePWV, as calculated by age and blood pressure, can predict CVD outcomes and mortality in the general population, independently of or beyond traditional CVD risk factors (8). In addition, several studies have shown that ePWV is robustly associated with cardiovascular events (e.g. coronary heart disease, stroke) (9–11). This suggests that ePWV may be an important predictor of the risk of developing disease and mortality. However, the long-term prognostic potential of ePWV in diabetic patients remains to be elucidated. Therefore, in this prospective study, we analyzed national sample data from the National Health and Nutrition Examination Survey (NHANES) to investigate whether arterial stiffness estimated by ePWV predicts all-cause and specific-cause mortality in individuals with DM.

## Methods

### Study design and population

This prospective cohort study was conducted using the national data from the NHANES from 1999 to 2014 and followed up until the end of December 2019. NHANES is a nationally representative survey of the civilian, non-institutional population in the United States. The survey includes interviews, physical examinations at home or mobile examination centers (MEC), and laboratory tests and is administered by the National Center for Health Statistics using a complex, stratified, multi-stage probability design. The survey is conducted every two years. Detailed sampling methods and the process of data collection have been published earlier (12). NHANES was implemented by the National Center for Health Statistics of the US Centers for Disease Control and Prevention (CDC) and approved by the National Center for Health Statistics Institutional Review Board. All participants provided written informed consent.

**Figure 1:**
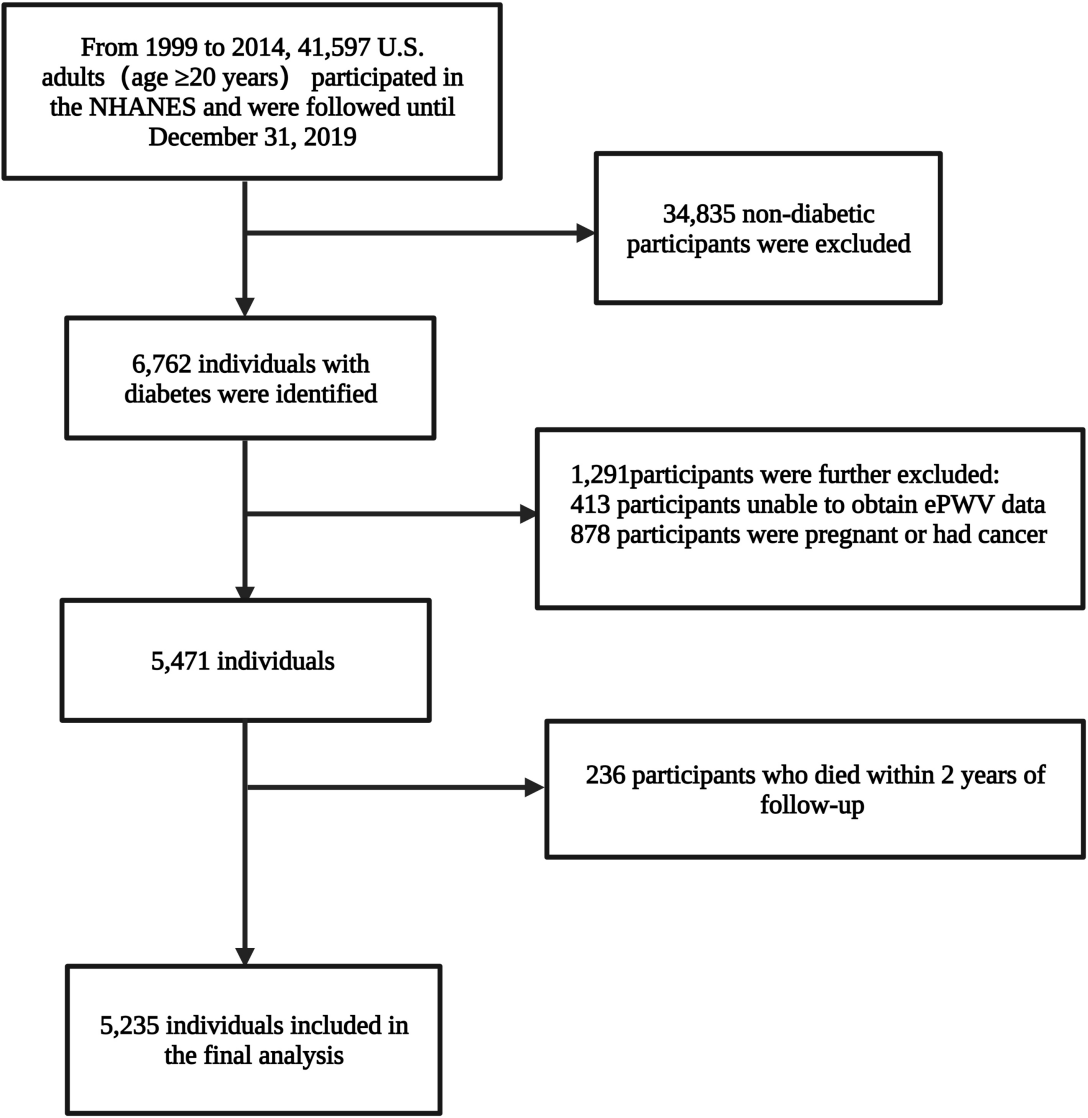
The study flow.

A total of 41,597 adults (age ≥20 years) from the USA participated in the eight survey cycles of the NHANES spanning 1999 to 2014. The diagnosis of diabetes was based on self-reported (as told by a medical doctor), use of insulin, or oral hypoglycemic drugs, fasting glucose≥7.0 mmol/L (126mg/dL), random blood glucose or two-hour oral glucose tolerance test blood glucose≥11.1 mmol/L or glycohemoglobin HbA1c≥6.5%. Finally, 6,762 individuals with diabetes were identified; ePWV data of 6,349 individuals were available; participants with cancer or pregnancy (n=878; self-reported) were further excluded. Concurrently, to reduce potential reverse causality bias, participants who died within two years of follow-up (n=236) were excluded. Ultimately, 5,235 individuals, representing 19,472,771 individuals with diabetes in the U.S., were included in the analysis (**Figure 1**).

### Calculation of estimated pulse wave velocity

The ePWV is calculated using the following formula (8).


$$\begin{array}{l} \text{ePWV}=9.587-0.402\times \text{age}+4.560\times 10^{-3}\times {\text{age}}^{2}-2.621\times 10^{-5}\times {\text{age}}^{2}\\ \times \text{mean blood pressure}\left(\text{MBP}\right)+3.176\times 10^{-3}\times \text{age}\times \text{MBP}-1.832\times 10^{-2}\times \text{MBP} \end{array}$$

In this formula, age is in years, and mean blood pressure (MBP) is calculated by diastolic blood pressure (DBP)+0.4×[systolic blood pressure (SBP)−DBP]. Participants were placed in a quiet sitting position for 5 min, and then a trained examiner used a uniform sphygmomanometer to measure the blood pressure. The blood pressure values were the average of at least three determinations. The technique for measuring blood pressure is as per the then latest recommendations of the American Heart Association Human Blood Pressure Determination using sphygmomanometers. Detailed information about the quality assurance & quality control process has been described in the Physician Section of the MEC Operations Manual (13).

### Outcome definition

The primary outcome of this article was all-cause mortality. The secondary outcomes were the nine cause-specific and residual-specific mortality reported by NHANES. Mortality for all-cause, cause- and residual-specific, was ascertained by linkage to the National Mortality Index until 31 December 2019. All-cause mortality included deaths from all the causes. Residual mortality is all the deaths except for the nine cause-specific mortality. Cause-specific mortality was coded by the International Classification of Diseases version 10 (ICD-10). The ICD codes for the nine cause-specific mortality are shown in **Table S1**.

### Other variables of interest

In this study, a set of some others variables were taken into consideration. Information on age, gender, race, level of education, family income, smoking and drinking status, history of diseases, and medication use was collected from home interviews and mobile examination centers utilizing standardized questionnaires.

Health status/medical history was obtained through a self-reported face-to-face interview. The questionnaire section is usually modelled on medical conditions questionnaire section of the U.S. National Health Interview Survey. These questions were asked by trained interviewers in the home using the computer assisted personal interviewing system. Take CVD for example. The interviewers would ask the participants the following questions: Have you ever been told by a doctor or other health professional that you have CVD?/how old were you when you were first told that you had CVD? In this paper, CVD was defined as a composite of diseases including coronary heart disease, congestive heart failure, heart attack, stroke, and angina. The participants had at least one of these conditions and were assumed to have CVD. Biochemical parameters were measured through a rigorous process, details of which can be found in the NHANES Procedures Manual for Laboratory/Medical Technologists (13). The participant’s blood pressure, weight, and height were measured at the mobile examination center. To facilitate data integration, we further classified the following variables:

i) Race: Non-Hispanic white people, non-Hispanic black people, Mexican Americans, or other races.
ii) Education levels: Less than 9th grade,9-11th grade/high school grade or equivalent, college graduate or above.
iii) Smoking status: It was classified as never (<100 cigarettes/lifetime), former (>;100 cigarettes/lifetime and absolutely no smoking now), and current (>;100 cigarettes/lifetime and currently smoking some days or every day) (14).
iv) Drinking status: It was categorized as never (<12 drinks/lifetime), former (≥12 drinks/lifetime but not drank in the last year), current light/moderate drinker (≤1 drink/day for women and ≤2 drinks/day for men in the past year), and current heavy drinker (>;1 drink/day for women and >;2 drinks/day for men in the past year) (15).

### Statistical analysis

In the data analysis process, appropriate weights (MEC weights) were selected to account for oversampling, nonresponse, and noncoverage and to provide nationally representative estimates. Detailed weighting methods are available on the NHANES website (https://wwwn.cdc.gov/nchs/nhanes/tutorials/Module3.aspx).

In demographic baseline characteristics, continuous variables are represented by means (standard error, SE), and categorical variables are represented by unweighted counts (weighted %). Levels of ePWV, as a continuous variable, were divided into quartiles, and Schoenfeld residuals verified the proportional hazard assumption. Kaplan-Meier analyses with a log-rank test were employed to analyze the cumulative survival of the diabetic population with different ePWV levels during the observation period. Survey-weighted Cox proportional hazards models were performed to assess the hazard ratios (HRs) and 95% confidence intervals (CIs) for the associations of ePWV with all-cause and cause-specific mortality. To reduce collinearity between age and ePWV, age is transformed into a categorical variable as it is directly involved in the calculation of ePWV. In addition, according to the classification of confounding covariates, they were progressively added to different models (Model 1-6). The fully adjusted model was conducted including all the baseline characteristics.

Subgroups analyses were conducted stratified by the following clinical characteristics: gender (male, female), age (<45,45-64,≥65 years), BMI (<30,≥30 kg/m^2^), diastolic blood pressure (<90, ≥90mmHg), systolic blood pressure (<140, ≥140mmHg), race (non-Hispanic white, non-Hispanic black, Mexican American, and others), asthma (no/yes), arthritis(no/yes), CVD (no/yes), hypertension (no/yes), chronic bronchitis (no/yes),and chronic kidney disease (CKD) (no/yes), and the P-values for interaction were obtained. Besides, the generalized additive model (GAM) was used to assess the dose-response relationship between ePWV and mortality visually, and the P-value for non-linear was obtained using the log-likelihood ratio test. As a complementary analysis, the relationship between MBP and the risk of mortality was assessed. GAM was performed using both log-transformed and untransformed methods. Log (HR) was converted to HR by taking antilog. A log (HR) of 0 and 1 implies a HR of 1 (not significant) and 2.71-fold, respectively (16). Threshold-effect analysis was employed to assess changes in the risk of all-cause mortality and other cause-specific mortality with increases in ePWV and MBP specific units. If a non-linear association was observed, the two-piecewise linear regression model was conducted to calculate the inflection point at which the ratio of ePWV and mortality changed markedly in the smoothed curve (17). For the missing variables, multiple imputations were performed based on the five-repetition predictive mean matching algorithm and Markov Chain Monte Carlo method, and the pooled results by the Cox regression model were considered as a form of sensitivity analysis (18). All analyses were conducted through the statistical packages R (http://www.R-project.org, The R Foundation) and EmpowerStats (Version 4.2.0, www.R-project.org, X&Y Solutions, Inc., Boston, MA). Two-tailed P-values less than 0.05 were considered statistically significant.

## Results

The current study analyzes the NHANES data from 1999 to 2014. A total of 5,235 individuals with DM were included. Their weighted mean age was 57.4 years, and 51.07% of them were male. The weighted overall demographic characteristics are mentioned in **Table 1**. During a median follow-up period of 115 months (interquartile range 81-155 months; 637,911 person-years), 1,604 all-cause deaths were recorded.

**Table 1:** Survey-weighted baseline characteristics of the diabetic population from NHANES 1999 to 2014 (N=5,235, representing 19,472,771 individuals).

ePWV	9.26 (0.04)
Demographic	
Age (years)	57.4 (0.29)
20-44	752 (18.37)
45-64	2,353 (48.54)
≥65	2,130 (33.09)
Male	2,677 (51.07)
Race	
Non-Hispanic white	1,780 (58.90)
Non-Hispanic black	1,388 (16.22)
Mexican American	1,195 (10.08)
Other races	872 (14.80)
Poverty income ratio	2.67 (0.04)
Parameters	
Body mass index	32.77 (0.17)
Waist	109.74 (0.40)
Systolic blood pressure	70.61 (0.32)
Diastolic blood pressure	71.37 (0.16)
Glycated hemoglobinA_1c_	7.23 (0.04)
Creatinine	83.96 (0.88)
Estimated glomerular filtration rate	85.75 (0.42)
Total cholesterol	5.03 (0.03)
High-density lipoprotein cholesterol	1.22 (0.01)
History of diseases	
Cardiovascular diseases	1,231 (22.37)
Chronic kidney disease	2,030 (36.15)
Asthma	734 (14.61)
Chronic bronchitis	392 (8.14)
Hypertension	3,777 (69.34)
Arthritis	2,160 (40.97)
Medication	
Antihypertensives	3,777 (69.34)
Glucose-lowering drugs	3,165 (59.25)
Lifestyle	
Smoking	
Never	2,600 (49.53)
Former	1,695 (32.14)
Current	934 (18.33)
Drinking	
Never	911 (16.44)
Former	1,504 (27.32)
Mild/Moderate	1,373 (31.64)
Heavy	1,107 (24.60)

Continuous variables are expressed as weighted mean (Standard error, SE). Categorical variables are expressed as counts (weighted %). NHANES, National Health and Nutrition Examination Survey. ePWV, Estimated pulse wave velocity.

### ePWV and all-cause mortality

**Figure 2:**
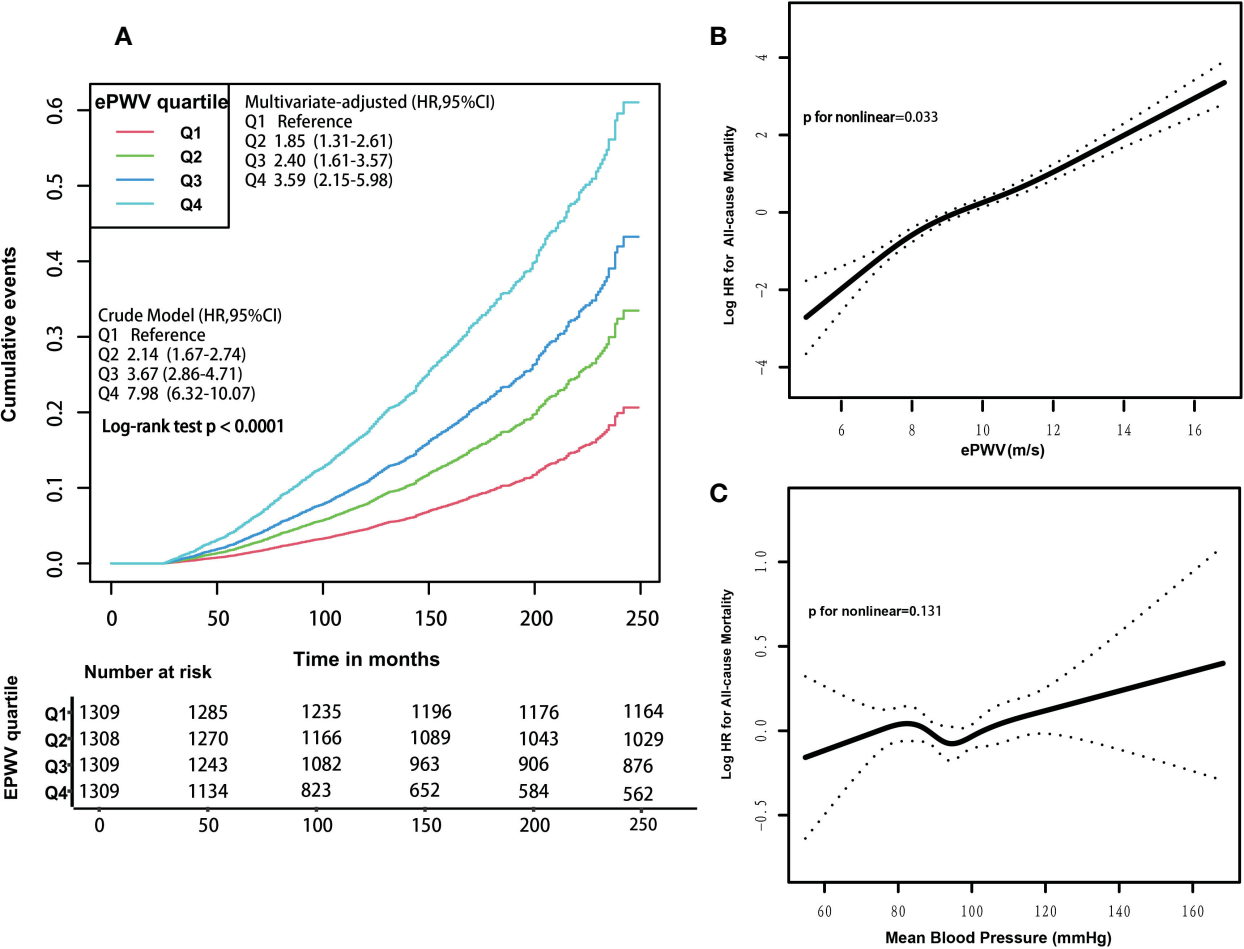
**(A)** Kaplan-Meier survival curves for all-cause mortality, according to ePWV quartile levels. Participants’ ePWV levels were grouped into quartiles, and crude and multivariate-adjusted model results were expressed as HRs and 95% CIs. Multivariate-adjusted model adjusted for the following variables: age, systolic blood pressure, diastolic blood pressure, gender, race, poverty income ratio, body mass index, waist, glycated hemoglobinA_1c_, total physical activity, creatinine, estimated glomerular filtration rate, total cholesterol, high-density lipoprotein cholesterol, cardiovascular diseases, chronic kidney disease, diabetes mellitus, chronic bronchitis, hypertension, Arthritis, antihypertensives and glucose-lowering drugs, smoking, drinking. HRs, hazard ratios. CIs, confidence intervals. **(B)** Associations between ePWV with the risk of all-cause mortality. Results were expressed as HRs and 95% CIs. In the generalized additive model, these following variables were fully adjusted: age, systolic blood pressure, diastolic blood pressure, gender, race, poverty income ratio, body mass index, waist, glycated hemoglobinA_1c_, total physical activity, creatinine, estimated glomerular filtration rate, total cholesterol, high-density lipoprotein cholesterol, cardiovascular diseases, chronic kidney disease, diabetes mellitus, chronic bronchitis, hypertension, Arthritis, antihypertensives and glucose-lowering drugs, smoking, drinking. HRs, hazard ratios. CIs, confidence intervals. **(C)** Associations between mean blood pressure levels with the risk of all-cause mortality. Results were expressed as HRs and 95% CIs. In the generalized additive model, these following variables were fully adjusted: age, gender, race, poverty income ratio, body mass index, waist, glycated hemoglobinA_1c_, total physical activity, creatinine, estimated glomerular filtration rate, total cholesterol, high-density lipoprotein cholesterol, cardiovascular diseases, chronic kidney disease, diabetes mellitus, chronic bronchitis, hypertension, arthritis, antihypertensives and glucose-lowering drugs, smoking, drinking. HRs, hazard ratios. CIs, confidence intervals.

A graded positive association was observed between increasing quartiles of ePWV and the risk of all-cause mortality, as shown by Kaplan-Meier curves, either in the crude (Q4 vs. Q1: 7.98; 95% CI:6.32 to 10.07; log-rank test P< 0.001) or multivariate-adjusted models (Q4 vs. Q1: 3.59; 95% CI:2.15 to 5.98; P<0.001) (**Figure 2A**). In the unadjusted Cox regression model, the risk of all-cause mortality increased by 46% (HR 1.46; 95% CI, 1.41 to 1.52; P<0.001) with an increase of 1m/s in ePWV (**Table 2**). Similarly, this hazard risk persisted after adjustment for age and blood pressure (HR 1.56; 95% CI, 1.46 to 1.66; P<0.001). Meanwhile, in the model fully adjusted for confounders, with every 1 m/s increase in ePWV, the risk of all-cause mortality increased correspondingly by 56% (HR 1.56; 95% CI, 1.44 to 1.69; P<0.001), which was comparable to the results after multiple imputations (HR 1.55; 95% CI, 1.46 to 1.66; P<0.001) (**Table S2**).

**Table 2:** Survey-Weighted Cox Proportional Hazard Results Examining the Association of ePWV on All-Cause and Cause-Specific Mortality in diabetes population from NHANES 1999 to 2014.

	Death	Unadjusted Model	Adjusted Model 1	Adjusted Model 2	Adjusted Model 3	Adjusted Model 4	Adjusted Model 5	Adjusted Model 6
ePWV, 1 m/s increase		HR (95%CI)	HR (95%CI)	HR (95%CI)	HR (95%CI)	HR (95%CI)	HR (95%CI)	HR (95%CI)
All-cause Mortality	1605	1.46 (1.41-1.52)^b^	1.56 (1.46-1.66)^b^	1.54 (1.42-1.65)^b^	1.51 (1.40-1.63)^b^	1.51 (1.39-1.64)^b^	1.51 (1.39-1.64)^b^	1.56 (1.44-1.69)^b^
**Specific causes**								
Cardiovascular disease	472	1.49 (1.39-1.58)^b^	1.55 (1.40-1.73)^b^	1.56 (1.39-1.74)^b^	1.47 (1.25-1.72)^b^	1.45 (1.22-1.72)^b^	1.45 (1.21-1.72)^b^	1.53 (1.27-2.83)^b^
Cerebrovascular disease	108	1.80 (1.65-1.98)^b^	1.79 (1.40-2.29)^b^	1.77 (1.36-2.30)^b^	1.83 (1.27-2.62)^a^	1.80 (1.26-2.58)^a^	1.83 (1.28-2.62)^b^	1.91 (1.30-2.80)^b^
Respiratory disease	71	1.51 (1.32-1.72)^b^	1.57 (1.17-2.10)^a^	1.46 (1.05-2.02)^a^	1.29 (0.89-1.87)^c^	1.36 (0.89-2.08)^c^	1.38 (0.91-2.09)^c^	1.65 (1.03-2.65)^a^
Alzheimer's disease	52	1.97 (1.75-2.21)^b^	2.27 (1.74-2.96)^b^	2.49 (1.92-3.22)^b^	2.31 (1.46-3.65)^b^	2.15 (1.29-3.85)^a^	2.15 (1.30-3.55)^a^	2.02 (1.15-3.57)^a^
Accidents	27	1.21 (0.98-1.50)^a^	1.26 (0.78-2.05)^c^	1.0 (0.55-1.83)^c^	1.11 (0.58-2.10)^c^	1.18 (0.60-2.33)^c^	1.18 (0.59-2.33)^c^	1.03 (0.56-1.91)^c^
Diabetes mellitus	173	1.31 (1.19-1.43)^b^	1.66 (1.32-2.08)^b^	1.51 (1.18-1.94)^a^	1.53 (1.15-2.04)^a^	1.56 (1.17-2.07)^a^	1.64 (1.22-2.20)^a^	1.56 (1.19-2.05)^a^
Renal disease	59	1.49 (1.22-1.81)^b^	1.34 (0.84-2.13)^c^	1.32 (0.79-2.22)^c^	1.24 (0.75-2.06)^c^	1.26 (0.79-2.03)^c^	1.26 (0.74-2.15)^c^	1.32 (0.73-2.37)^c^
Caner	232	1.32 (1.22-1.42)^b^	1.31 (1.12-1.55)^b^	1.35 (1.12-1.62)^a^	1.49 (1.21-1.85)^b^	1.47 (1.18-1.84)^b^	1.47 (1.18-1.84)^c^	1.54 (1.22-1.96)^b^
Influenza and pneumonia	108	1.78 (1.45-2.17)^b^	1.76 (1.19-2.62)^a^	1.72 (1.09-2.71)^a^	1.73 (1.10-2.73)^a^	1.73 (1.05-2.84)^a^	1.71 (0.98-2.97)^c^	1.98 (1.10-3.55)^a^
Residual (all other causes)	374	1.45 (1.36-1.54)^b^	1.54 (1.39-1.73)^b^	1.55 (1.37-1.75)^b^	1.51 (1.27-1.79)^b^	1.47 (1.23-1.76)^b^	1.47 (1.23-1.76)^b^	1.54 (1.27-1.86)^b^

Respiratory diseases indicated all deaths from chronic lower respiratory diseases. Renal disease indicated all deaths from nephritis, nephrotic syndrome, and nephrosis.

a indicates p-value<0.05;

b indicates p-value<0.001;

c indicates p-value≥0.05.

**Model 1** adjust age (20-44,45-64,≥65years), systolic blood pressure (continuous), diastolic blood pressure (continuous).

**Model 2** adjust Model 1 plus other demographic variables including gender (male, gender), race (non-Hispanic white, non-Hispanic black, Mexican American, and other races), and poverty income ratio (continuous).

**Model 3** adjusted Model 2 plus other parameters including body mass index (continuous), waist (continuous), glycated hemoglobinA_1c_ (continuous), total physical activity(continuous), creatinine (continuous), estimated glomerular filtration rate (continuous), total cholesterol (continuous), and high-density lipoprotein cholesterol (continuous).

**Model 4** adjusted Model 3 plus history of diseases including cardiovascular diseases (yes/no), chronic kidney disease (yes/no), diabetes mellitus (yes/no), chronic bronchitis (yes/no), hypertension (yes/no), and Arthritis (yes/no).

**Model 5** adjusted Model 4 plus medication including antihypertensives(yes/no)and glucose-lowering drugs (yes/no).

**Model 6** adjusted Model 5 plus lifestyle variables including smoking (never, former, and current), and drinking (never, former, mild/moderate, and heavy).

**Figure 3:**
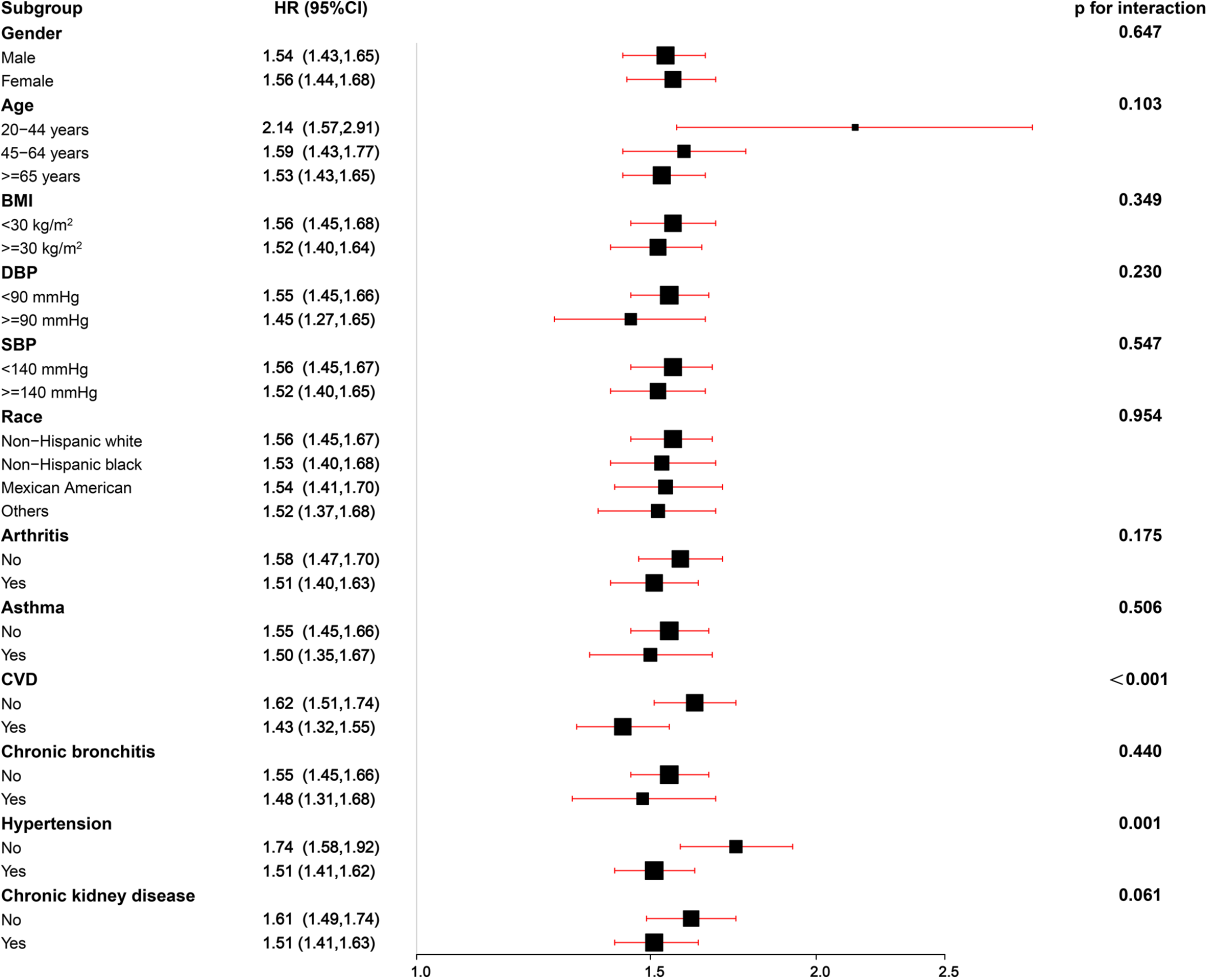
Stratified analyses of the associations (hazard ratios, 95% CIs) between ePWV values and all-cause mortality in patients with DM. HRs were fully adjusted by the following covariates including age, systolic blood pressure, diastolic blood pressure, gender, race, poverty income ratio, body mass index, waist, glycated hemoglobinA_1c_, total physical activity, creatinine, estimated glomerular filtration rate, total cholesterol, high-density lipoprotein cholesterol, cardiovascular diseases, chronic kidney disease, diabetes mellitus, chronic bronchitis, hypertension, arthritis, antihypertensives and glucose-lowering drugs, smoking, drinking.

In the subgroup analyses, the positive association between ePWV and risk of all-cause mortality was consistent across most strata (p=0.061-0.954), and the risk of all-cause mortality increased by 43%-114% with the increase of 1m/s ePWV (**Figure 3**). However, interactions were observed in CVD and hypertension groups (P for interaction<0.05), suggesting that the strength of the association between ePWV and all-cause mortality risk may differ in these groups. The positive association remained between ePWV and risk of all-cause mortality regardless of grouping.

### ePWV and cause-specific mortality

In the unadjusted Cox regression model, there was a positive association between ePWV and the risk of cause-specific mortality (**Table 2**). With increasing levels of 1m/s ePWV, the increased risk of special-cause mortality ranged from 21-97%. However, after adjustment for age and blood pressure levels, ePWV remained positively associated with most cause-specific mortality except for renal disease and accident-specific mortality. Meanwhile, after fully adjusting for confounding, each 1m/s increase was associated with a 53% (HR 1.53; 95% CI, 1.27 to 2.83), 91% (HR 1.91; 95% CI, 1.30 to 2.80), 65% (HR 1.65; 95% CI, 1.03 to 2.65), 102% (HR 2.02; 95% CI, 1.15 to 3.57), 56% (HR 1.56; 95% CI, 1.19 to 2.05),54% (HR 1.54; 95% CI, 1.22 to 1.96), 98% (HR 1.98; 95% CI, 1.10 to 3.55), and 54% (HR 1.54; 95% CI, 1.27 to 1.86) increase in CVD, cerebrovascular disease, respiratory disease, Alzheimer’s disease, DM, cancer, influenza and pneumonia, and residual-specific mortality, respectively (**Table 2**). Likewise, comparable results were obtained after multiple imputations (**Table S2**).

### Dose-dependent relationship between ePWV levels and risk of all-cause and cause-specific mortality

A positive nonlinear association existed between ePWV levels and the risk of all-cause mortality (P for non-linear=0.033) (**Figure 2B**). In addition, threshold effect analysis showed a 2.1-fold (HR 3.10; 95% CI 1.54 to 6.25; P=0.002) increase in the risk of all-cause mortality for every 1 m/s increase in ePWV when ePWV was<7.2 m/s. There was a 54% (HR 1.54; 95% CI 1.44 to 1.64; P<0.001) increase in the risk of all-cause mortality for every 1 m/s increase in ePWV when ePWV was ≥7.2 m/s (**Table 3**). A linear relationship between ePWV and the risk of all-cause mortality was also reported, as the P for non-linear was very close to 0.05. For each 1 m/s increase in ePWV, the risk of all-cause mortality increased by 55% (HR 1.55; 95% CI 1.44 to 1.65; P< 0.001).

**Table 3:** Threshold-effect analysis on ePWV, MBP and all-cause and cause-specific mortality.

Inflection-point	HR	p-value	p for non-linear		HR	p-value	p for non-linear
	ePWV (1m/s increase)				Mean blood pressure (5 mmHg increase)		
**All-cause Mortality**			0.033	**All-cause Mortality**	1.01 (0.99-1.03)	0.302	0.131
<7.2	3.10 (1.54-6.25)	0.002					
≥7.2	1.54 (1.44-1.64)	<0.001					
Cause-specific Mortality							
**Cardiovascular diseases**			0.038	**Cardiovascular diseases**			0.029
<7.09	7.23 (1.25-41.72)	0.027		<97mmHg	0.95 (0.89-1.02)	0.166	
≥7.09	1.54 (1.36-1.74)	<0.001		≥97mmHg	1.08 (1.01-1.16)	0.018	
**Cerebrovascular diseases**			0.001	**Cerebrovascular disease**	1.06 (0.96-1.17)	0.282	0.157
<7.94	Inf. (0.0-Inf.)	0.550		**Cancer**	0.92 (0.87-0.98)	0.010	0.443
≥7.94	2.03 (1.51-2.74)	<0.001		**Influenza and pneumonia**	1.16 (1.00-1.34)	0.049	0.168
**Cancer**			0.030	**Respiratory diseases**	0.93 (0.83-1.04)	0.20	0.288
<8.72	1.71 (1.41-2.06)	<0.001		**Alzheimer's disease**	1.01 (0.88-1.16)	0.890	0.479
≥8.72	0.67 (0.26-1.70)	0.396		**Diabetes mellitus**			0.050
**Influenza and pneumonia**			0.046	<94 mmHg	1.22 (1.03-1.44)	0.021	
<12.83	1.34 (1.06-1.69)	0.013		≥94 mmHg	0.97 (0.87-1.09)	0.590	
≥12.83	3.70 (2.22-6.19)	<0.001		**Residual mortality**	1.04 (0.99-1.09)	0.085	0.140
**Respiratory diseases**			0.011				
<11.37	2.32 (1.46-3.69)	<0.001					
≥11.37	0.98 (0.57-1.72)	0.957					
**Alzheimer's disease**	2.31 (1.55-3.43)	<0.001	0.069				
**Diabetes mellitus**	1.47 (1.19-1.82)	<0.001	0.353				
**Residual mortality**	1.47 (1.27-1.69)	<0.001	0.093				

Respiratory diseases indicated all deaths from chronic lower respiratory diseases. ePWV, estimated pulse wave velocity. MBP, mean blood pressure.

HRs for ePWV have been fully adjusted for the following variables: age (20-44, 45-64, ≥65years), systolic blood pressure, diastolic blood pressure, gender, gender, race, poverty income ratio , body mass index, waist, glycated hemoglobinA_1c_, creatinine, estimated glomerular filtration rate, total cholesterol, high-density lipoprotein cholesterol, cardiovascular diseases (yes/no), chronic kidney disease (yes/no), diabetes mellitus(yes/no), chronic bronchitis (yes/no), hypertension (yes/no), Arthritis(yes/no), antihypertensives(yes/no), glucose-lowering drugs (yes/no), smoking (never, former, and current), and drinking (never, former, mild/moderate, and heavy).

HRs for mean blood pressure have been fully adjusted for the following variables: age (continuous), gender, race, poverty income ratio , body mass index, waist, glycated hemoglobinA_1c_, creatinine, estimated glomerular filtration rate, total cholesterol, high-density lipoprotein cholesterol, cardiovascular diseases (yes/no), chronic kidney disease (yes/no), diabetes mellitus(yes/no), chronic bronchitis (yes/no), hypertension (yes/no), Arthritis(yes/no), antihypertensives(yes/no), glucose-lowering drugs (yes/no), smoking (never, former, and current), and drinking (never, former, mild/moderate, and heavy).

Similarly, non-linear positive associations were observed between ePWV levels and risk of CVD (P for non-linear=0.038), cerebrovascular disease (P for non-linear=0.001), cancer (P for non-linear=0.03), influenza and pneumonia (P for non-linear=0.046), and respiratory disease mortality (P for non-linear=0.011) (**Table 3**; **Figure S1–S7**). However, ePWV levels were linearly positively associated with the risk of Alzheimer’s disease (P for non-linear=0.069), DM (P for non-linear=0.353), and residual mortality (P for non-linear=0.093) (**Table 3**).

In addition, we additionally analyzed the association of MBP with mortality. MBP was not significantly associated with the risk of all-cause and most cause-specific mortality (all p value > 0.05), except for CVD and DM mortality (**Table 3**; **Figure 2C**; **Figure S1–S7**). MBP was nonlinearly associated with the risk of CVD mortality (P for non-linear=0.029); when MBP was<97 mmHg, MBP was not significantly associated with the risk of CVD mortality (HR 0.95; 95% CI 0.89 to 1.02; P=0.166); however, when MBP was ≥97 mmHg, the risk of CVD mortality increased by 8% (HR 1.08; 95% CI 1.01 to 1.16; P=0.018) for every 5 mmHg increase in MBP (**Table 3**). Similarly, we observed a non-linear relationship between MBP and the risk of DM mortality (P for non-linear=0.050); when MBP was<94 mmHg, there was a corresponding 22% (HR 1.22; 95% CI 1.03 to 1.44; P=0.021) increase in the risk of DM mortality for every 5 mmHg increase in MBP (**Table 3**).

## Discussion

The current findings suggest that ePWV is an independent risk factor for all-cause and most cause-specific mortality in patients with DM, except for accident- and renal disease-specific mortality. ePWV was nonlinearly and positively associated with the risk of all-cause mortality. In addition, among cause-specific mortality, ePWV was nonlinearly positively associated with the risk of CVD, cerebrovascular disease, cancer, influenza and pneumonia, and respiratory disease mortality. It showed linearly positive association with the risk of Alzheimer’s disease, DM, and residual-specific mortality. These positive associations were independent of age and blood pressure levels. In addition, MBP was not significantly associated with the risk of all-cause and most cause-specific mortality and was only non-linearly associated with mortality from CVD and DM. Within a specific range of MBP (MBP_CVD_≥97mmHg, and MBP _Diabetes_<94mmHg), MBP was positively associated with the risk of CVD and diabetes mortality.

Several studies have recently shown that ePWV can predict mortality risk in the general population and patients with CVD or with an increased risk of CVD (7, 8, 19–21).ePWV is also suggested to be associated with subsequent risk of mortality and cardiovascular morbidity, independent of systemic coronary risk assessment and the Framingham risk score. Hefferman et al. analyzed a cohort of NHANES from 1999 to 2006 and found that in the general population with no CVD, with an increase in ePWV at 1 m/s, the risk of all-cause and CVD mortality increased by 50% and 47%, respectively (8).In addition, Laugesen et al. followed 25,066 patients with stable angina for 8.5 years and found a 13% increase in the risk of all-cause mortality with a1 m/s increase in ePWV levels (20). In a multicenter study of 107,599 healthy individuals, Vishram-Nielsen et al. observed a 13% increase in the risk of all-cause mortality for every 1 m/s increase in ePWV levels but no significant association with the risk of CVD mortality (21). They also showed a considerable variation in the additive prognostic information for high and low ePWV between European countries, suggesting a strong cohort dependence in the association between ePWV and mortality. Current studies have focused on the assessment of ePWV with all-cause and CVD mortality, while other cause-specific mortality has not been well studied. In the present study, we found a 55% increase in all-cause mortality for every 1 m/s increase in ePWV in the US diabetic population. In addition, the risk of mortality increased from 53% to 102% with a 1m/s increase in ePWV for most cause-specific mortality, except for accident and renal disease. Compared with previous studies, ePWV was associated not only with a higher risk of all-cause mortality but also with a higher risk of most cause-specific mortality, suggesting that “cohort-generality” and “cohort-dependence” of ePWV on mortality risk may objectively present. Therefore, there is a need to evaluate this in a larger number of models and populations. These results also suggest that atherosclerosis is prevalent throughout the pathophysiology of death and drives the onset of death. Thus, ePWV may serve as a proxy for vascular age, reflecting the aging of the whole organism and providing additional risk assessment for specific mortality in a way that traditional risk factors cannot.

Advanced age is an essential factor in natural human aging. Arterial stiffness progresses with aging, which directly affects both blood pressure and pulse pressure. The ePWV is calculated based on age and blood pressure levels. Therefore, in general, the older the person, the higher the ePWV value. In the multivariate Cox regression model, we adjusted multiple factors: age and blood pressure, traditional risk factors (such as BMI, blood lipid levels), poor lifestyle habits, history of chronic disease and medication use, and other potential risk factors, with consistent results before and after multiple imputation. Moreover, stratified analyses provided further evidence of the robustness of these findings. Interestingly, in our analysis of MBP, we observed no significant association for MBP with all-cause mortality and most cause-specific mortality, except for CVD and diabetes mortality. These findings provide further evidence that the association of ePWV with mortality independent of blood pressure levels captures additional risks of mortality, while solidly low levels of MBP also confer additional benefits.

Several factors can trigger the development of arterial stiffness, including endothelial dysfunction, reduced nitric oxide bioavailability, inflammation, oxidative stress, and hormone deficiency (e.g., estrogen) (22–25). In addition, metabolic abnormalities, poor lifestyle and diet, age, and genetic factors can lead to structural remodeling of the blood vessels (26–28) resulting in arterial stiffness. Arterial stiffness is strongly associated with cause-specific mortality. Several mechanisms may be involved. Changes in atherosclerosis include thickening, fibrosis, fragmentation, and loss of elastin fibers, which can lead to structural changes in the arterial wall and stiffening (29); on the other hand, arterial stiffness can make the intima more susceptible to damage and atherosclerosis (30), leading to cardiovascular and cerebrovascular events. In addition, DM affects endothelial function (31), which increases the risk of arterial stiffness, and arterial stiffness also affects the function of the peripheral circulation, increasing the incidence of diabetic complications. Arterial stiffness is positively correlated with the severity of airway obstruction (32, 33).Hence, the more severe the arterial stiffness, the worse is the lung function, increasing the incidence or even worsening respiratory diseases. On the other hand, respiratory diseases are often associated with systemic inflammation, sympathetic activation, and chronic hypoxic states, which further contribute to arterial stiffness (24).The link between arterial stiffness and cancer may be due to many co-existing risk factors, such as smoking and inflammation. Meanwhile, the use of anti-cancer drugs can in turn lead to arterial stiffness (34).Besides, arterial stiffness and the development of Alzheimer’s disease may be associated with small vessel disease, stroke, and brain atrophy (35). In conclusion, the relationship between arterial stiffness and mortality from these specific causes is usually bidirectional, with different risk factors interacting.

### Perspectives

Our findings indicated that ePWV was positively and nonlinearly associated with the risk of all-cause mortality, whereas MBP was not significantly associated with the risk of all-cause mortality. Although the level of ePWV is highly dependent on blood pressure and age, ePWV can predict the risk of mortality in DM patients independently of these two parameters. The results of this study highlight the added benefit of steadily lowering blood pressure in people with DM, rather than lowering blood pressure per se. Furthermore, the measurement of arterial stiffness has a positive impact on the assessment of mortality risk in patients with DM. Recently, it has been shown that there is a strong association between ePWV and CfPWV (r=0.7) (36). Similarly, a recent study by Heffen et al. showed that ePWV is associated with established indicators of vascular ageing such as carotid thickness, carotid stiffness and augmentation dilatation index (37). Therefore, ePWV may be a useful tool in the assessment of vascular aging. In conditions where cfPWV cannot be measured, ePWV could be considered as an assessment of arterial stiffness for mortality risk stratification. Currently, there is no reasonable quantitative method to distinguish between normal and abnormal ePWV levels. Future studies could consider grouping by age and blood pressure level to quantify the predictive value of ePWV as a parameter for mortality risk.

### Strength and limitation

As far as we know, this is the first study to examine ePWV and the risk of all-cause and cause-specific mortality in a cohort with DM, complementing the current field of study. Additionally, this study is a large prospective cohort design with a long follow-up period and a nationally representative sample, which helps to generalize our findings. Inevitably, this study has a few limitations. First, due to the design of this observational study, causal effects, and exclusion of the impact of residual confounders are not well obtained. Second, the cohort observed here is a diabetic population from the US and does not distinguish between diabetes subtypes. The results cannot be generalized to other specific populations. Third, the participants’ diagnosis of chronic disease history was primarily self-reported and may have been subjectively potentially biased. Fourth, due to the limitations of the current data, we are not able to make a definitive classification of glucose-lowering drugs. Although adjustments have been made for glucose-lowering drugs, the effect of differences in the type of glucose-lowering drug on the results remains unclear.

## Conclusion

In the diabetic population, ePWV is independently associated with all-cause and most cause-specific mortality risks. ePWV may be a useful tool for assessing mortality risk.

## Data availability statement

The datasets presented in this study can be found in online repositories. The names of the repository/repositories and accession number(s) can be found in the article/**Supplementary Material**.

## Ethics statement

The studies involving human participants were reviewed and approved by National Health and Nutrition Examination Survey. The patients/participants provided their written informed consent to participate in this study.

## Author contributions

CL and SC designed this topic. CL and HP drafted, analyzed, and interpreted this study. CL, HP, FK, SY, QS, DL and SC critically reviewed the study. All authors read and approved the submitted manuscript. All authors contributed to the article and approved the submitted version.
