# Supplementary material for: Association of arterial stiffness with all-cause and cause-specific mortality in the diabetic population: A national cohort study
Source: Front Endocrinol (Lausanne). 2023 Mar 8;14:1145914. doi: 10.3389/fendo.2023.1145914 (PMC10031114; doi:10.3389/fendo.2023.1145914)
Supplement: Supplementary file 1 [file DataSheet_1.docx]

| **Table S1. ICD-10 code for the nine Cause-specific mortality** | |
| --- | --- |
| **CVD** | **I00-I09, I11, I13, I20-I51** |
| **Cerebrovascular diseases** | **I60-I69** |
| **Accidents (unintentional injuries)** | **V01-X59, Y85-Y86** |
| **Chronic lower respiratory diseases** | **J40-J47** |
| **Diabetes mellitus** | **E10-E14** |
| **Influenza and pneumonia** | **J09-J18** |
| **Alzheimer's disease** | **G30** |
| **Nephritis, nephrotic syndrome, and nephrosis** | **N00-N07, N17-N19, N25-N27** |
| **Cancer** | **C00-C97** |

| **Table S2. Survey-weighted Cox proportional hazards results examining the association of ePWV with all-cause and cause-specific mortality in diabetic population from NHANES 1999 to 2014 (follow-up to 2019).** | | | |
| --- | --- | --- | --- |
| **Outcomes** | **Death** | **Pooled results*** | |
| **ePWV, 1 m/s increase** |  | **HR (95%CI)** | **P value** |
| All-cause Mortality | **1605** | **1.55 (1.46-1.66)** | **＜0.001** |
| **Specific causes** |  |  |  |
| Cardiovascular disease | 472 | **1.57 (1.37-1.79)** | **＜0.001** |
| Cerebrovascular disease | 108 | **1.83 (1.39-2.42)** | **＜0.001** |
| Respiratory diseases | 71 | **1.89 (1.29-2.75)** | **0.001** |
| Alzheimer's disease | 52 | **2.22 (1.46-3.39)** | **＜0.001** |
| Renal disease | 59 | **1.14 (0.75-1.71)** | **0.541** |
| Accidents | 27 | **1.63 (0.94-2.82)** | **0.082** |
| Diabetes mellitus | 173 | **1.57 (1.27-1.94)** | **＜0.001** |
| Caner | 232 | **1.46 (1.21-1.77)** | **＜0.001** |
| Influenza and pneumonia | 108 | **1.69 (1.04-2.73)** | **0.033** |
| Residual (all other causes) | 374 | **1.44 (1.26-1.66)** | **＜0.001** |

**Respiratory diseases indicated all deaths from chronic lower respiratory diseases. Renal disease indicated all deaths from nephritis, nephrotic syndrome, and nephrosis.**

**HR indicates the change in corresponding risk of mortality for each 1m/s increase in ePWV.**

*** represents the pooled results of the multiple imputations (5 data sets**)

The HRs have been fully adjusted for age (20-44, 45-64, ≥65years), systolic blood pressure (continuous), diastolic blood pressure (continuous), gender (male, gender), and poverty income ratio (continuous), body mass index (continuous), waist (continuous), total physical activity (continuous), creatinine (continuous), estimated glomerular filtration rate(continuous), total cholesterol (continuous), high-density lipoprotein cholesterol (continuous), cardiovascular diseases (yes/no), chronic kidney disease (yes/no), diabetes mellitus(yes/no), chronic bronchitis (yes/no), hypertension (yes/no), Arthritis(yes/no), antihypertensives(yes/no), glucose-lowering drugs (yes/no), smoking (never, former, and current), and drinking (never, former, mild/moderate, and heavy).

**Figure S1. Associations between ePWV (left) and mean blood pressure levels (right) with the risk of CVD mortality. In the generalized additive model, the confounders have been fully adjusted.**

**
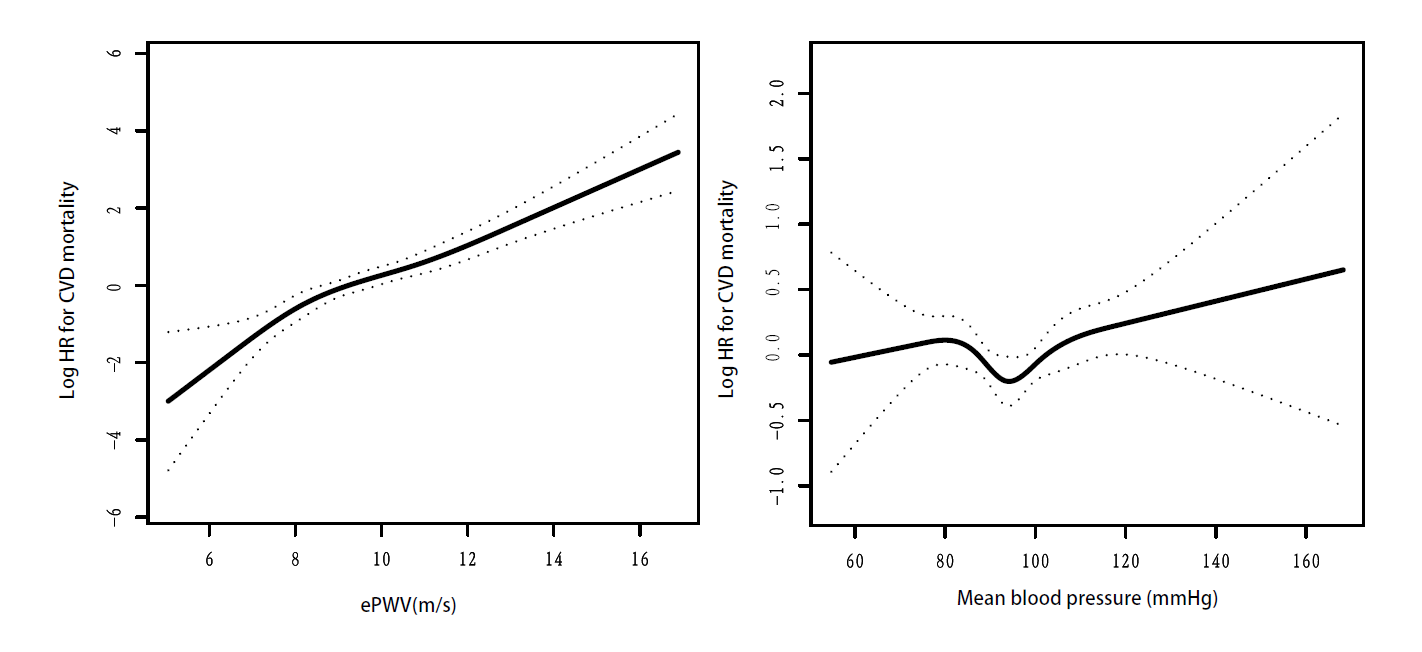
**


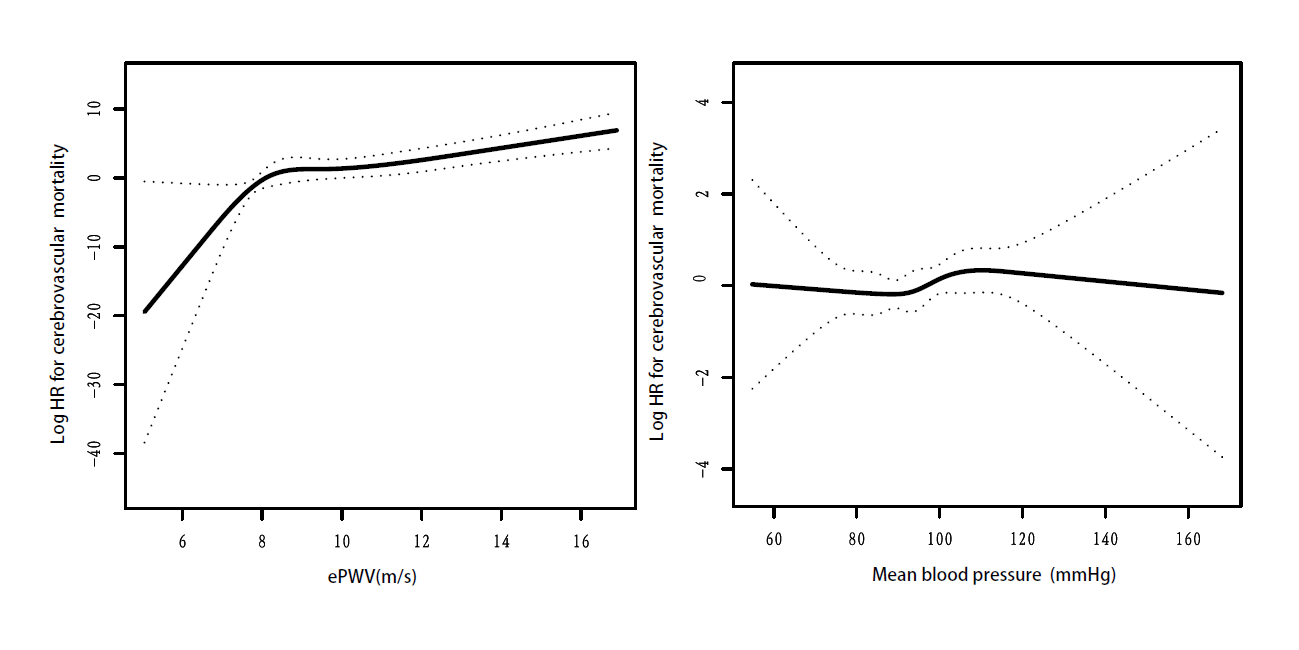
**Figure S2. Associations between ePWV (left) and mean blood pressure levels (right) with the risk of cerebrovascular mortality. In the generalized additive model, the confounders have been fully adjusted.**


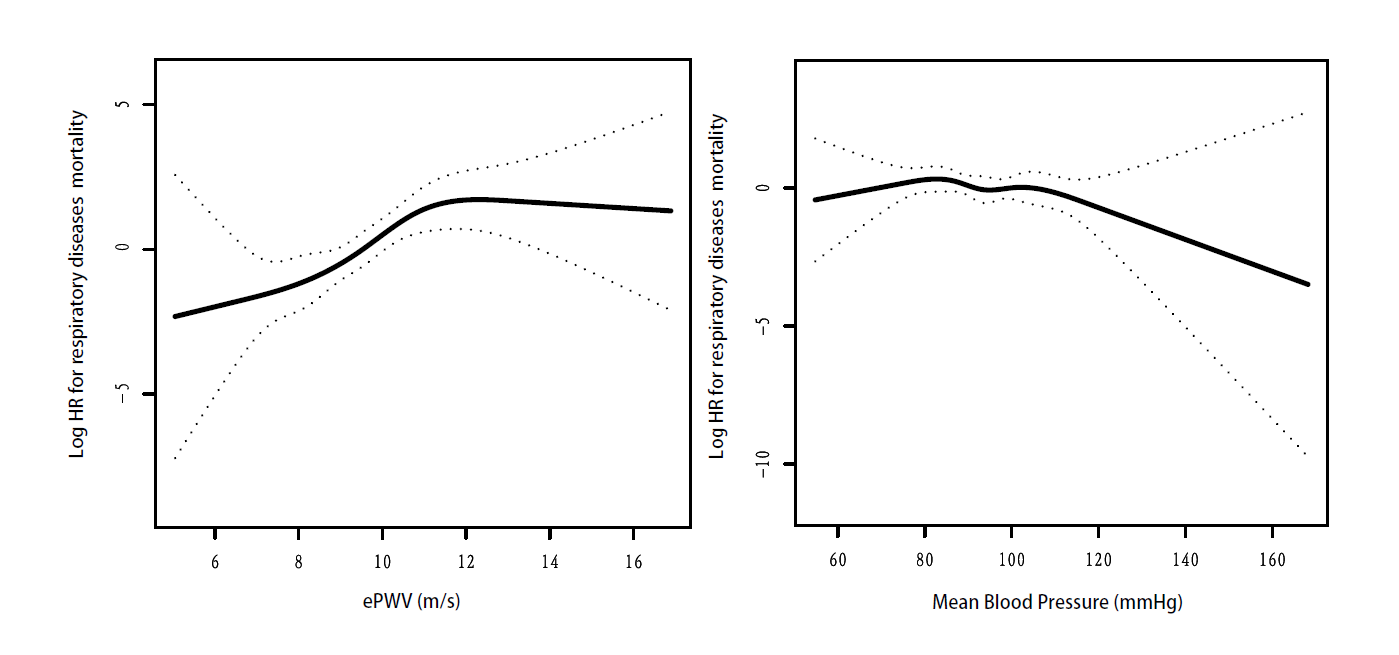
**Figure S3. Associations between ePWV (left) and mean blood pressure levels (right) with the risk of respiratory diseases mortality. In the generalized additive model, the confounders have been fully adjusted.**

**
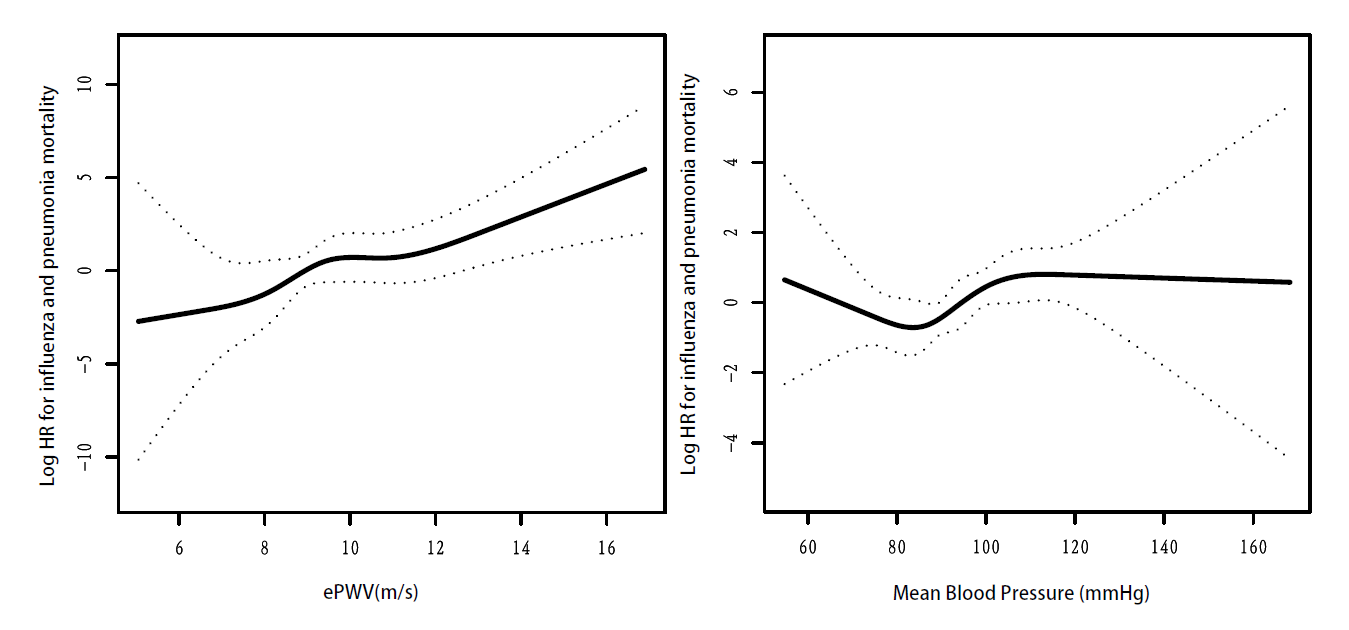
Figure S4. Associations between ePWV (left) and mean blood pressure levels (right) with the risk of influenza and pneumonia mortality. In the generalized additive model, the confounders have been fully adjusted.**

**Figure S5. Associations between ePWV (left) and mean blood pressure levels (right) with the risk of diabetes mellitus mortality. In the generalized additive model, the confounders have been fully adjusted.**


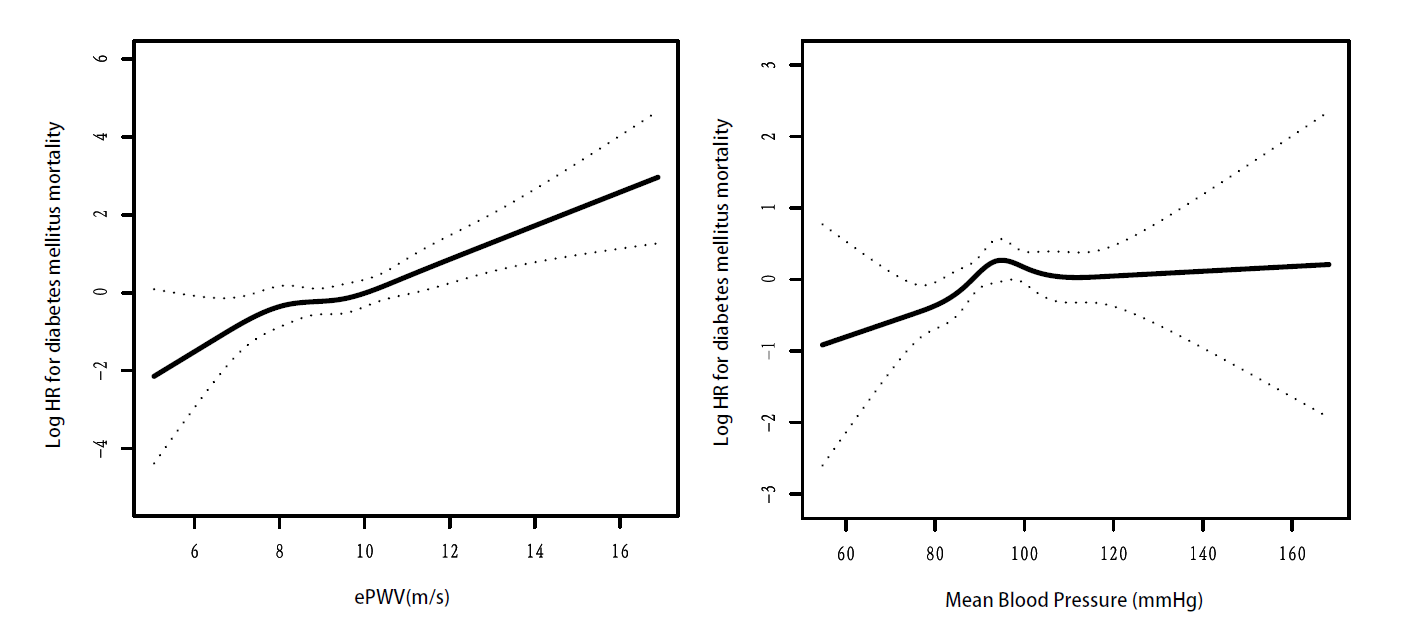


**Figure S6. Associations between ePWV (left) and mean blood pressure levels (right) with the risk of Alzheimer’s disease mortality. In the generalized additive model, the confounders have been fully adjusted.**


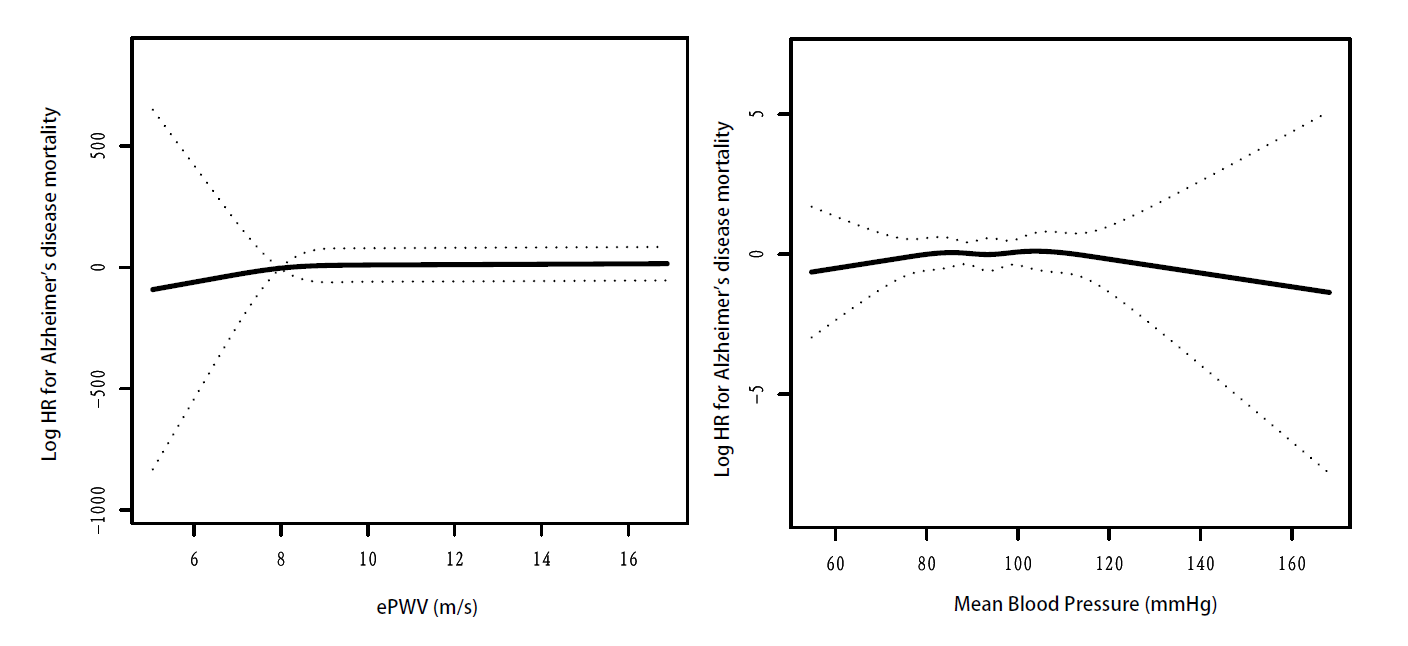


**Figure S7. Associations between ePWV (left) and mean blood pressure levels (right) with the risk of residual-specific mortality. In the generalized additive model, the confounders have been fully adjusted.**

**
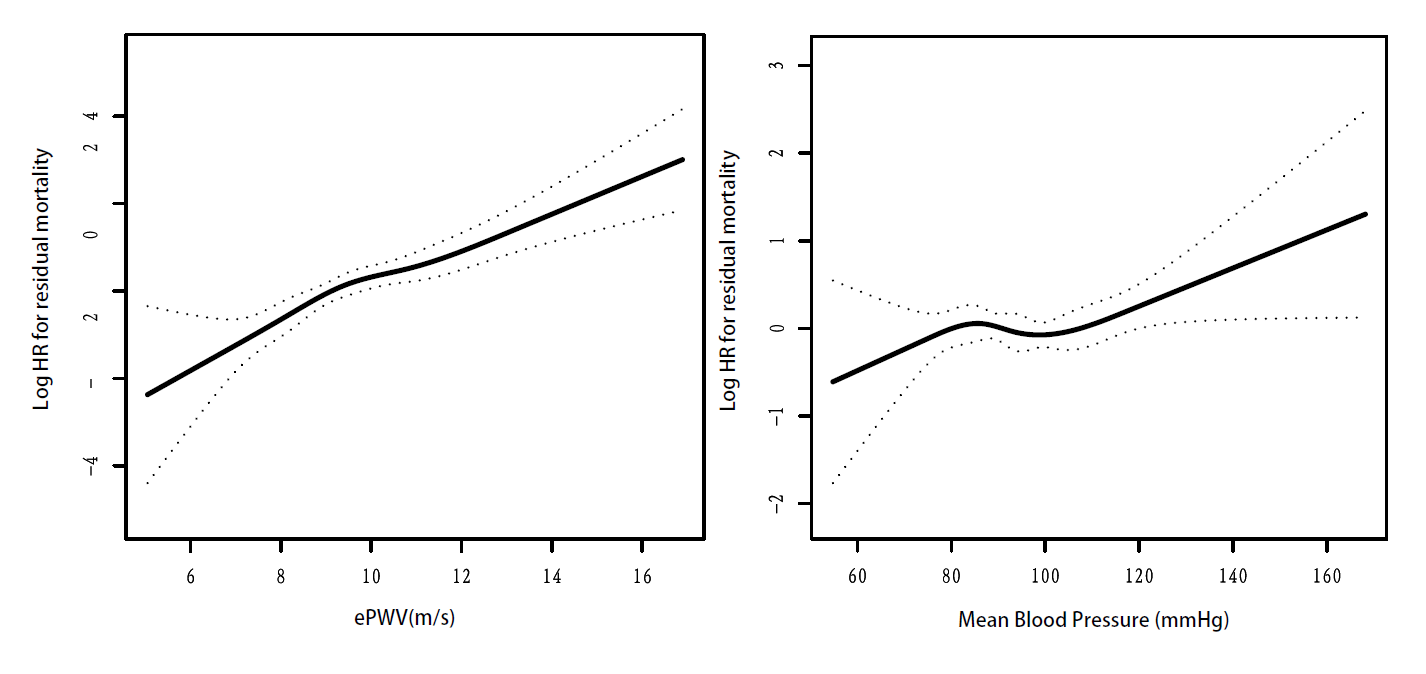
**
